# Surgical resection of advanced intrathoracic tumors through a combination of the hemiclamshell and transmanubrial approaches

**DOI:** 10.1007/s00595-024-02838-6

**Published:** 2024-05-06

**Authors:** Yudai Miyashita, Eriko Fukui, Hiroto Ishida, Toru Kimura, Takashi Kanou, Naoko Ose, Soichiro Funaki, Yasushi Shintani

**Affiliations:** Department of General Thoracic Surgery, Osaka University Graduate School of Medicine, 2-15 Yamadaoka, Suita, Osaka 565-0871 Japan

**Keywords:** Thymoma, Lung cancer, Hemiclamshell approach, Transmanubrial osteomuscular sparing approach

## Abstract

**Purpose:**

The hemiclamshell (HCS) approach provides a comprehensive view of the anterior mediastinum, whereas the transmanubrial osteomuscular sparing approach (TMA) allows sufficient exposure of the cervico-thoracic transition. We assessed the effectiveness and the outcomes of the combined HCS plus TMA approach to resect thoracic malignant tumors.

**Methods:**

We reviewed five patients with thoracic malignant tumors invading the thoracic outlet who underwent surgery using an HCS and TMA approach between 2018 and 2021.

**Results:**

The preoperative diagnosis was myxofibrosarcoma, lung cancer, thymic cancer, thymoma, and neurofibromatosis type1 in one patient each, respectively. Cardiovascular reconstruction was done on the aortic arch in two patients, on the descending aorta in one, and on the superior vena cava in one, combined with resection of the vagus nerve in three patients, of the phrenic nerve in two, and of vertebra in one, with overlap in some cases. The TMA was added because all patients required dissection of the periphery of the subclavian artery, and two had tumor extension to the neck. Macroscopic complete resection was achieved in four patients. There was no postoperative mortality.

**Conclusion:**

The combination of the HCS and TMA approaches at the same operation provides a comprehensive view of the mediastinum, lung, and cervico-thoracic transition and allows safe access to the thoracic great vessels and subclavian vessels.

## Introduction

Intrathoracic tumors are difficult to detect and often become large and invade the surrounding area. Surgical resection is the choice of treatment and complete resection improves the prognosis significantly [1–3]. Therefore, the surgical approach is important. In particular, the approach to the apex of the lung is controversial as there are various ways to approach the apex of the chest. The hemiclamshell (HCS) approach, developed by Masaoka et al., comprises a partial median sternotomy and antero-lateral thoracotomy [4]. This approach enables access to the mediastinum, apex of the chest, and thoracic great vessels for advanced lung cancer [5]. It is also useful for lung resection performed for advanced thymic malignancy with hilar invasion by providing multiple access paths to the tumor and hilum [6]. However, it is difficult to obtain a view that extends into the profound cervico-thoracic transition, such as the subclavian vessels via an HCS approach alone. Grunenwald reported the transmanubrial osteomuscular sparing approach (TMA), which comprises the L-shaped resection of the manubrium and the first cartilage section and preserves the clavicle and sternoclavicular joint [7]. TMA was selected as the surgical approach for tumors located in the anterior compartment or involving the subclavian vessels. Tumors that invade the vertebral artery in the periphery of the subclavian artery have also been reported to be eligible for the TMA [8]. This retrospective study reviews five patients who underwent surgery via a combination of an HCS approach and TMA for a thoracic malignant tumor invading the thoracic outlet, to evaluate its advantages.

## Methods

### Patients

This study was conducted in accordance with the Declaration of Helsinki (revised in 2013) and approved by the Institutional Review Board of Osaka University (Approval No. 18518). The need for individual consent was waived as this was a retrospective analysis and data were accessed after masking the patients’ identity. The subjects were five patients with thoracic malignant tumors invading thoracic outlet who underwent surgical resection via the combination of an HCS approach and the TMA between 2018 and 2021.

### Surgical technique

All patients with a mediastinal tumor or a lung cancer underwent chest computed tomography (CT) and some also underwent chest magnetic resonance imaging (MRI) to establish the degree of invasion of the surrounding organs. Surgical resection was planned immediately when a tumor was seen to be only in contact with or with slight invasion of a large vessel. If complete resection was not expected because of severe tumor invasion of the surrounding organs, a tumor biopsy using CT-guided or video-assisted thoracic surgery (VATS) was performed, and then induction therapy was scheduled according to the diagnosis. Following induction therapy, resectability was judged again based on chest CT findings, and then surgical resection was planned within 4–8 weeks after the completion of therapy. Surgery was indicated for thymoma patients with disseminated lesions, but not for lung cancer patients with disseminated lesions, who continued to receive chemoradiotherapy.

An HCS approach was selected for patients with a mediastinal tumor, or a lung cancer suspected of invasion extending to the aorta or main pulmonary artery and mediastinum, including the superior vena cava (SVC), atrial wall, or apical thoracic dome, as described previously [5]. When the tumor extended deep in the cervico-thoracic transition, such as infiltration to distal subclavian vessels, then TMA was added.

**Fig.1:**
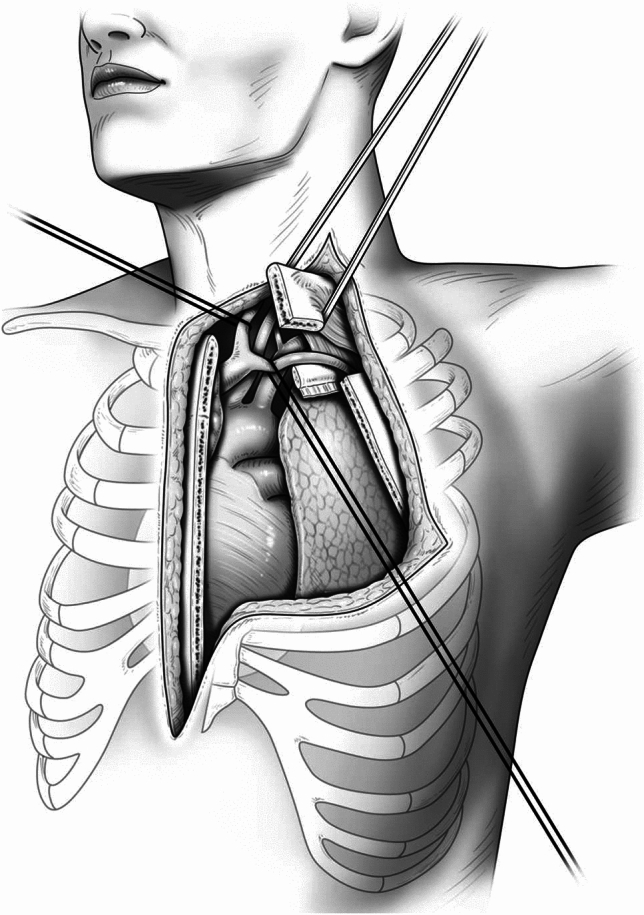
The representative surgical view offered by the hemiclamshell approach with a transmanubrial osteomuscular sparing approach, which allowed a wide antero-lateral view of the mediastinal vascular complex and profound cervico-thoracic transition. *HCS* hemiclamshell, *TMA* transmanubrial osteomuscular sparing approach

We reported previously on the surgical procedure for an HCS approach [1, 5, 6]. The patient is placed in a semi-lateral decubitus position and secured firmly to the surgical bed for its rotation. Before beginning this approach, thoracoscopic inspection is generally performed to confirm tumor invasion extending to the pulmonary hilum and rule out dissemination in patients with thymic cancer. A cutaneous incision with an L or inverted L shape is used to avoid a T-shaped incision. Thoracic access is then obtained with an upper median sternotomy and a fourth or fifth intercostal thoracotomy. For patients with deep pleural dissemination, a fifth intercostal thoracotomy is performed. When treating patients who require cardiopulmonary bypass for resection of the tumor, full sternotomy should be performed first to secure the right side of the heart [3]. For additional TMA, an ipsilateral collar incision is performed to obtain a more comprehensive view and prevent skin tightening, and 25% of the superoexternal part of the manubrium is sectioned through an L-shaped incision, thereby preserving the sternoclavicular articulation. Subsequently, the first costal cartilage is resected and the costoclavicular ligament is divided, which allows full lateral access to the subclavian vessels [7]. Figure 1 shows a representative surgical view offered by the HCS approach plus TMA. A wide antero-lateral view of the mediastinal vascular complex and the profound cervico-thoracic transition can be obtained.

## Results

**Fig. 2:**
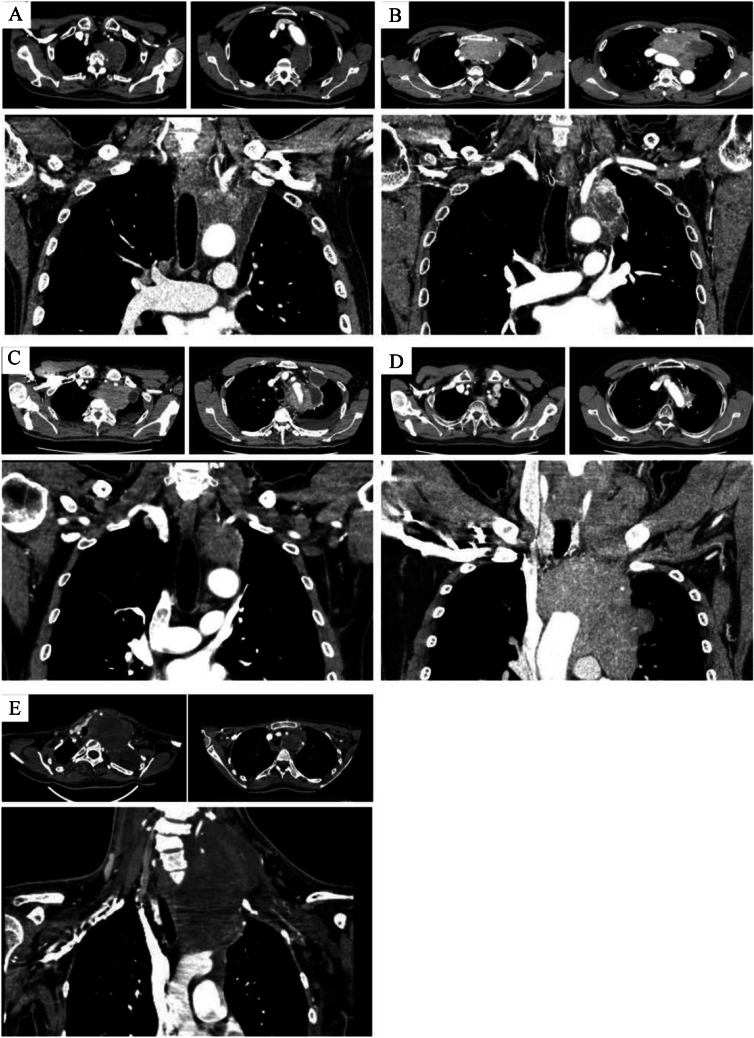
**A**. Computed tomography (CT) images showing a tumor with suspected invasion of the lung, aorta, vertebra and subclavian artery (Case 1). **B**. CT images showing a tumor with suspected invasion of the lung, hilar, and aorta (Case 2). **C**. CT images showing a tumor with suspected invasion of the lung, aorta, subclavian artery, and phrenic nerve (Case 3). **D**. CT images showing a tumor with suspected invasion of the lung, aorta, and subclavian artery, with possible extension to the neck (Case 4). **E**. CT images showing a tumor with suspected invasion of the left subclavian artery and subclavian vein, with possible extension to the neck (Case 5). *SCA* subclavian artery, *SCV* subclavian vein

Table 1 summarizes the clinical characteristics of the five patients who underwent a radical resection via an HCS approach combined with the TMA. The median age at the time of surgery was 56 years (range, 25–60 years). The preoperative diagnosis was myxofibrosarcoma, lung cancer, thymic cancer, thymoma and neurofibromatosis type1 in one patient each, respectively. The median size of the tumors was 77 mm (range, 52–120 mm). Four of the five patients underwent preoperative therapy, as chemotherapy in three and embolization of the left VA in one. Figure 2 shows the chest CT findings. Infiltration of both the lung and aorta was suspected in four patients and SCA infiltration was seen in all patients (Table 1).

**Table 1:** Clinical characteristics of the patients

Case	1	2	3	4	5
Age/gender	70/M	56/M	62/M	47/M	25/F
Tumor size(mm)	77	52	72	118	120
Preoperative diagnosis	Myxofibrosarcoma	Lung ca.	Thymic ca.	Thymoma	Neurofibromatosis type1
Clinical stage	T3N0M0	T4N0M0	T4N0M0	T4N2M0	T4N0M2
Preoperative therapy	(–)	CBDCA, PTX, RT	CBDCA, PTX	ADOC	Embolisation of the left VA
Clinically infiltrated organs					
Lung	●	●	●	●	
Hilar				●	●
Aorta	●	●	●	●	
SCA	●	●	●	●	●
Others	Vertebra		Phrenic nerve	Extension to the neck	Left vertebral artery

*lung ca* lung cancer, *thymic ca* thymic cancer, *CBDCA* Carboplatin, *PTX* Paclitaxel, *ADOC* Adriamycin, cisplatin, vincristine, and cyclophosphamide, *RT* radiation therapy, *VA* vertebral artery, *SCA* subclavian artery

**Fig. 3:**
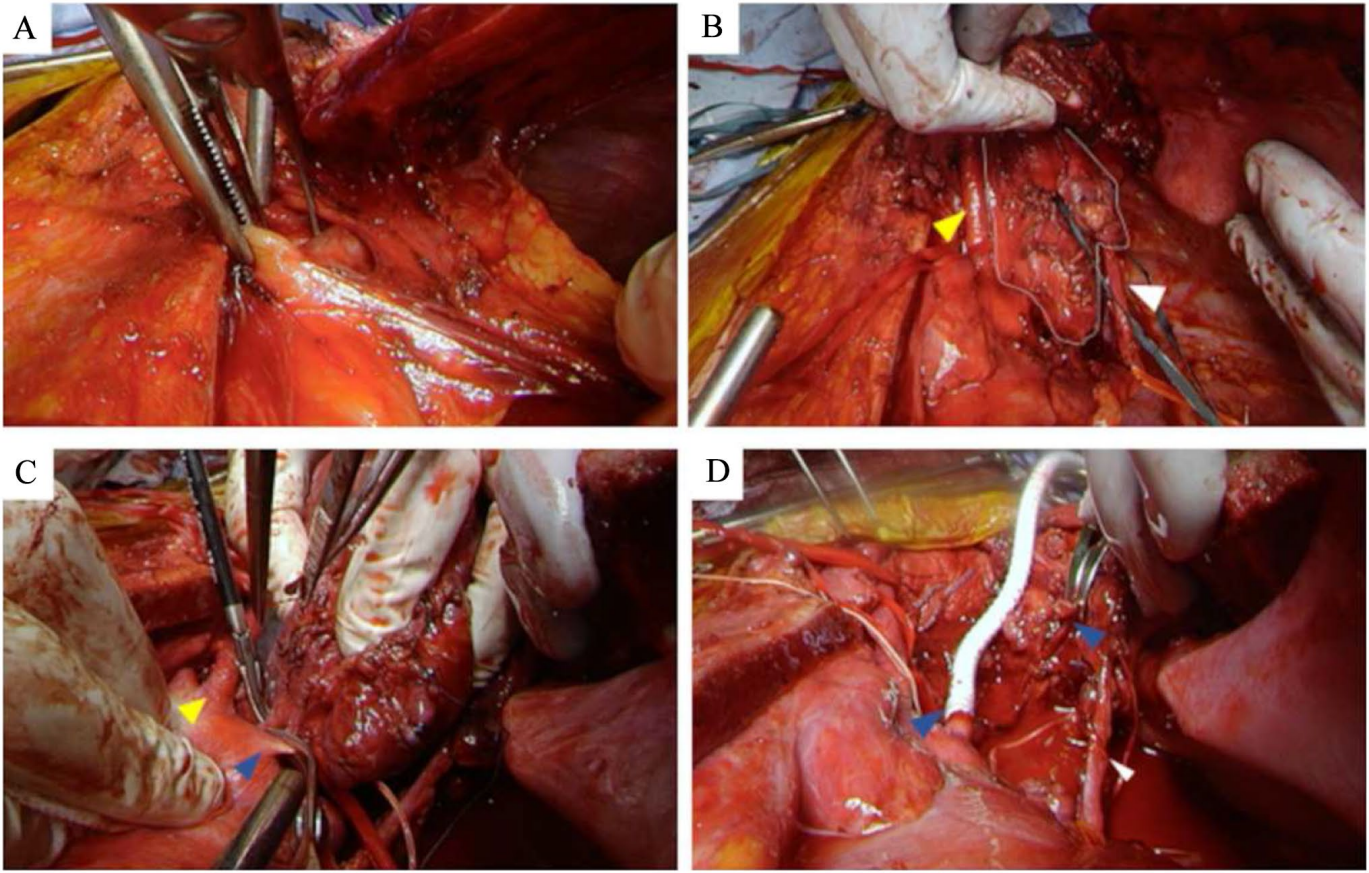
Surgical view of the hemiclamshell approach only. **A** Surgical view of the hemiclamshell approach plus the transmanubrial osteomuscular sparing approach. The anterior compartment can be seen. Yellow arrow head; Left common arotid artery, White arrow head; phrenic nerve, White line; main tumor. **B** Access to the subclavian artery was enabled. Yellow arrow head; Left common carotid artery, Blue arrow head; subclavian artery. **C** View of the reconstruction of the subclavian artery. Blue arrow head; subclavian artery, White arrow head; phrenic nerve. *HCS* hemiclamshell, *TMA* transmanubrial osteomuscular sparing approach, *SCA* subclavian artery, *CCA* common carotid artery, *VA* vertebral artery

Figure 3 shows a representative surgical view provided by the combination of the HCS approach combined with the TMA (Case 5). The peripheral to the subclavian artery (SCA) can be identified. As shown in Table 2, four patients underwent left upper lobectomy. The organs resected included the vagus nerve in three patients, the aortic arch in two, the phrenic nerve in two, the descending aorta in one, the left SCA in one, the left BCV in one, the left SCV in two, and a vertebra in one, with overlap in some cases. TMA was added because all patients required dissection to the periphery of the SCA and two had tumor extension to the neck. Both patients with phrenic nerve resection underwent diaphragmatic plication through an HCS approach to prevent paradoxical diaphragmatic movement. The median operative time was 684 min (range, 608–823 min) and blood loss was 3660 mL (range, 760–5720 mL). Pneumonia developed in two patients and nerve complications developed in two, including Horner syndrome in one and recurrent nerve paralysis in one. One patient suffered cerebral infarction, managed with conservative treatment. The median postoperative hospital stay was 37 days (range, 19–94 days). There were no complications related to the surgical approach and there was no postoperative mortality. Macroscopic complete surgical resection was achieved in four patients, but this could not be achieved in Patient 1 as a white nodule was found on the chest wall intraoperatively. The intraoperative rapid pathological diagnosis was inflammation, but both the tumor itself and the white nodule on the chest wall were diagnosed as mesothelioma postoperatively. The median follow-up period was 16.5 months. At the time of this analysis, one patient had died. Of the remaining four surviving patients, one with mesothelioma and one with thymoma were alive with relapse.

**Table 2:** Operative procedure and results

Case	Resected organs				Resection	Complications	Relapse	DFS	Survival	Outcome
	Lung	Aorta	Nerve	Others	Status			(Mo)	(Mo)	
1	LUL	Descending		Lt SCV, vertebra(Th2-4)	R2	Horner syndrome		0	9	AWD
2	LUL	Arch	Vagus		R0	Pneumonia		7	7	NED
3	LUL	Arch	Phrenic, vagus		R0	Recurrent nerve paralysis		24	24	NED
4	LUL		Phrenic, vagus	lt BCV	R0	Pneumonia, cerebral infarction	BONE	12	21	AWD
5				lt SCA, SCV	R0	None	BONE	3	9	DOD

*LUL* left upper lobectomy, *SCA* subclavian artery, *SCV* subclavian vein, *BCV* brachiocephalic vein, *DFS* disease free survival, *AWD* alive with disease, *NED* no evidence of disease, *DOD* died of disease, *Mo* months.

## Discussion

Mediastinal tumors or lung cancers often present as large masses, and their delayed detection may be associated with extension into the neck. Surgical resection is the primary treatment for large tumors in the cervico-thoracic junction, but attaining R0 resection is crucial for achieving long-term survival [1–3]. Therefore, selecting the appropriate surgical approach is imperative [3]. While median sternotomy is the standard technique for accessing the mediastinum, it poses challenges in reaching the upper thorax, lower thoracic cavity, and lung hilum [9]. An alternative approach known as an HCS approach allows for better visualization of the pulmonary hilum and enables access to the diaphragm [4]. The TMA is preferred when tumors are suspected of invading the cervico-thoracic transition or SCA. The TMA allows for comprehensive surgical exposure, *en bloc* extended resection without compromising bone and muscle integrity, and favorable functional outcomes [7]. A recent report showed enhanced visualization of the vertebral body through refinements to the classic TMA [10]. Nevertheless, these techniques are limited in their ability to approach the hilar region or tracheal bifurcation, and the application of the TMA is primarily restricted to tumors located in the upper mediastinum and neck. VATS and lateral thoracotomy incisions can be used in conjunction, but they may not provide an adequate field of view when dealing with large tumors that have invaded the surrounding structures [11–13]. Spaggiari reported successful outcomes using a combination of the TMA and median sternotomy, which allowed for bilateral dissection of the subclavian and brachiocephalic veins, as well as access to the thoracic compartments [13]. Zhong described a “reverse L surgical approach” involving a median sternotomy and the TMA for the resection of large tumors at the cervico-thoracic transition. They reported no mortalities or cases of delayed wound healing, and all patients were discharged from hospital [14].

Incorporating the TMA into an HCS approach achieved a broader visual field than the previously reported method of combining the TMA with a median sternotomy. As shown in Fig. 1, the integration of the TMA into an HCS approach provided visibility from the subclavian artery to the branching vertebral artery. This approach is beneficial when the tumor extends proximal to the vertebral artery and enhances visualization of the brachial plexus, recurrent nerve, and transverse nerves, ultimately improving postoperative quality of life. While there have been reports of the TMA combined with additional posterolateral incisions or VATS, these approaches necessitate intraoperative repositioning [11, 12, 15, 16]. Conversely, our approach obviates the need for repositioning and offers a potentially superior field of view without changing the patient's position, surpassing the findings of these reports. In our institution, five patients underwent tumor resection via TMA performed in conjunction with an HCS approach. Consequently, two patients underwent successful reconstruction of the left subclavian artery with aortic arch replacement, and complete resection was achieved in four, despite their advanced stage of disease. Notably, there were no complications attributable to the surgical approach, indicating its advantages with appropriate patient selection. Even if there is extensive invasion, the combination of the TMA and an HCS approach facilitated the safe attainment of complete resection.

## Conclusion

An HCS approach used with the TMA provided good operative fields for both the cervico-thoracic transition and the hilum of the lung. No complications such as delayed wound healing occurred, suggesting that this technique is suitable for tumors that extend deep into the neck and require hilar manipulation.
